# Multimodality Imaging of COVID-19 Using Fine-Tuned Deep Learning Models

**DOI:** 10.3390/diagnostics13071268

**Published:** 2023-03-28

**Authors:** Saleh Almuayqil, Sameh Abd El-Ghany, Abdulaziz Shehab

**Affiliations:** 1 Department of Information Systems, College of Computer and Information Sciences, Jouf University, Sakaka 72388, Saudi Arabia; snmuayqil@ju.edu.sa (S.A.); saabdelwahab@ju.edu.sa (S.A.E.-G.); 2 Department of Information Systems, Mansoura University, Mansoura 35516, Egypt

**Keywords:** COVID-19, deep learning, healthcare, transfer learning, multimodality

## Abstract

In the face of the COVID-19 pandemic, many studies have been undertaken to provide assistive recommendations to patients to help overcome the burden of the expected shortage in clinicians. Thus, this study focused on diagnosing the COVID-19 virus using a set of fine-tuned deep learning models to overcome the latency in virus checkups. Five recent deep learning algorithms (EfficientB0, VGG-19, DenseNet121, EfficientB7, and MobileNetV2) were utilized to label both CT scan and chest X-ray images as positive or negative for COVID-19. The experimental results showed the superiority of the proposed method compared to state-of-the-art methods in terms of precision, sensitivity, specificity, F1 score, accuracy, and data access time.

## 1. Introduction

The first confirmed COVID-19 case, according to the World Health Organization (WHO) reports, was in the central Chinese city of Wuhan on 8 December 2019. COVID-19 was reported as an epidemic in January 2020. From that date onwards, new confirmed cases were reported each day and the COVID-19 virus spread to every continent. According to the WHO, the number of confirmed COVID-19 virus cases is more than 416,614,050 and the number of deaths is more than 5,844,095 [1]. COVID-19 has become a global health crisis and the WHO has declared it a major pandemic. Understanding how the disease spreads and determining how undetected and undocumented cases contribute to the transmission of the virus are major challenges. Although COVID-19 vaccines are available on the market, there is still an inevitable demand for smart healthcare systems for the general population—and, especially, the elderly—so that the expected shortage in doctors in the health sector during the crisis can be promptly addressed. With the spread of COVID-19 to numerous countries in the world, considering the increases in the number of people infected and the number of deaths from day to day, it has become mandatory to diagnose and identify this COVID-19 virus.

All over the world, the COVID-19 virus remains a threat to the economies of countries and the health of people. It has been proven that the disease is transmitted from one person to another and, therefore, delays in discovering the disease lead to the spread of infection through interactions between the healthy and infected patients [2,3,4].

The test to verify a person’s infection with the virus is often implemented by taking samples from the patient’s throat, sputum, or nasopharynx to analyze the PCR of the viral RNA. However, one of the limitations of such tests is their low accuracy [5,6,7,8]. The diagnosis of COVID-19 based on laboratory tests is costly, laborious, time-consuming, and involves a complicated manual process [9]. Therefore, it is recommended that this type of test be replaced with chest CT images, which could be used as one type of early investigative test [3]. The challenge of laboratory testing with CT image analysis is that it demands a radiology expert and takes a significant amount of time. One solution for this challenge is to save valuable time for medical professionals by using an automated analysis system.

Thus, the early diagnosis of COVID-19 would be invaluable in containing the disease outbreak. However, as the diagnosis of COVID-19 based on laboratory tests still displays a low positive rate, as well as being costly, laborious, time-consuming, and manually complicated [9], suggestions have emerged from specialists recommending the use of radiography screening as a primary tool for checking for COVID-19. With increased numbers of infected patients on a daily basis, especially in the epidemiological setting, the bottleneck for this method is the requirement for a large number of expert X-ray specialists to interpret the CT images. Moreover, the similar and overlapping patterns of lung diseases make it difficult for radiologists to point out these slight differences [10]. As a result, there is an urgent need to develop intelligent systems to aid radiologists with fast and accurate results.

Artificial intelligence (AI) has made great strides in recent years. Deep learning and the accompanying innovations offer radiologists a chance to change the radiology scene and improve survival rates. Deep learning neural system models have been applied to a few imaging tasks to date, including image segmentation, classification, and object detection. Deep learning techniques are unique compared to classic AI strategies, which are the significant ones. The latter employ feature extraction strategies in preparation for the computation, while deep learning techniques familiarize themselves with the image information without the requirement for feature extraction. Deep learning provides promising solutions for the analysis of medical images in future applications. With the recent advances in machine learning and artificial intelligence applications, especially in the medical field and including medical image processing, artificial intelligence has become a promising tool that can change diagnosis methods. Deep learning networks, as an artificial intelligence tool, have proven successful in image classification with their unique characteristics of being able to learn image representations automatically and mapping features onto continuous vectors that are subsequently used for predictions. AI applications in radiology [11] are driven by the idea that medical images are sets of data that can be computed by a machine to extract useful information from the properties of the image [12].

Thus, this paper presents a novel technique for the detection of the COVID-19 virus early on based on X-ray and CT chest radiography image analysis using deep learning algorithms. This methodology can be basically classified as image analysis as it comprises steps such as image acquisition, image preprocessing, feature extraction, and the employment of different classifiers. This paper’s contributions can be outlined as follows: (1) We built promising, fine-tuned DL models capable of diagnosing chest X-ray and CT images that showed improvements in their precision, sensitivity, specificity, F1-scores, accuracy, and data access time. The models for COVID-19 detection were trained and their performances were evaluated using novel samples in order to contribute to the control of the epidemic. (2) The proposed system will reduce the pressure on the national healthcare budget by reducing the cost of the physical tests performed in clinical laboratories. It has been reported that the cost of a PCR test is about USD 120–130 [13]. (3) For radiologists, with the spread of COVID-19, a daily flood of CT images need to be analyzed; the proposed techniques can act as an aid for radiologists (regardless of the level of experience) by reducing not only the time required to diagnose CT scans but also the pressure on the radiologist. (4) The disease can be localized by using a Grad-CAM algorithm that visualizes the infected areas of the lungs in chest X-ray and CT images.

The remainder of the paper is laid out as follows: Section 2 contains a literature review; Section 3 presents the proposed methodology; Section 4 contains the experimental results and discussion; and, finally, conclusions are drawn in Section 5.

## 2. Literature Review

The attention the COVID-19 virus has received from researchers has led to an enormous number of publications. In this section, most of the recently developed systems applying deep learning techniques to COVID-19 detection are explored. Barstugan et al. [14] proposed a coronavirus classification technique for CT images based on machine learning methods. The dataset, consisting of patients from Italy, one of the most endemic regions, comprised 618 images, including 219 images from COVID-19 patients, 224 images from influenza-A viral pneumonia patients, and 175 images from healthy cases. Chowdhury [15] trained four convolutional neural networks (CNNs) to classify CT images into two classes: normal and COVID-19 pneumonia. Linda et al. [9] proposed COVID-net, which is a deep CNN that can recognize and identify the COVID-19 disease from CT images. Shuai et al. [16] adapted the Inception transfer learning model to detect COVID-19 in CT images. Ghoshal et al. [17] presented a Bayesian deep learning classifier to estimate model uncertainty using the transfer learning method with COVID-19 X-ray images. The proposed system differs from the others in tracking disease progression through a deep analysis of the periodical changes in the CT images from the same patient, making it possible to modify treatment and help achieve improved results for patients. It also utilizes an efficient parameterized transfer learning model and smart data augmentation.

Rahimzadeh et al. [18] proposed a linked CNN dependent on the Xception and ResNet50V2 models to characterize COVID-19 cases from chest X-rays. The created framework utilized a dataset that contained 180 images of COVID-19 patients, 6054 images of pneumonia patients, and 8851 images of typical individuals. For each of the eight preparation stages, 633 pictures were selected. The test results exhibited 99.56% precision with 80.53% of COVID-19 cases. Fan et al. [19] proposed a multi-kernel attention network to analyze chest X-ray images from COVID-19 patients. Their model has three stages: a feature extraction stage followed by two parallel multi-kernel-size attention modules and, finally, the classification stage. The experimental results demonstrated improved performance in COVID-19 detection and an accuracy of 98.2%.

Loey et al. [20] presented a generative adversarial network (GAN) using deep learning to analyze COVID-19 from chest X-rays. Their study utilized the three pre-prepared models AlexNet, GoogleNet, and RestNet18. Apostolopoulos et al. [21] presented an exchange learning technique with a CNN for the analysis of COVID-19 cases using chest X-rays. The framework can detect COVID-19 images using five main models: VGG19, Inception, MobileNet, Xception, and Inception-ResNetV2. VGG19 was chosen as the fundamental learning model and it showed 93.48% accuracy. To detect COVID-19 patients, Panwar et al. [22] presented a binary image classification task. The input data were classified using a fine-tuned VGG model. Mishra et al. [23] used deep CNN-based image classification models to differentiate COVID-19 instances using chest CT scan images. Song et al. [24] employed a linear classifier to extract semantic features from CT scans. Jaiswal et al. [2] used a DenseNet201-based deep transfer learning (DTL) model to identify patients with COVID-19. The proposed model uses its own training weights to extract features from the ImageNet dataset. Silva et al. [25] proposed CovidNet, an efficient, voting-based technique for analyzing COVID-19 patterns in CT images.

Allioui et al. [26] proposed a multi-agent deep learning model for enhancement of COVID-19 CT image segmentation. Their proposal was based on multi-agent deep reinforcement learning (DRL), which utilizes a modified version of the Deep Q-Network. Khan et al. [27] proposed a COVID-19 detection method for CT images using deep learning, entropy-controlled optimization, and parallel feature fusion techniques. Their method mainly depends on the AlexNet and VGG16 models. The features are extracted and fused using a parallel positive correlation approach. Then, the entropy-controlled firefly optimization method is employed to select the optimal features. Their best achievement was an accuracy rate of 98%. Rehman et al. [28] proposed a framework for the detection of COVID-19 disease and 14 other types of chest diseases. They employed a convolutional neural network architecture with a soft-max classifier. Then, transfer learning was applied in order to extract deep features, which provided results similar to classic machine learning classification methods. Guo et al. [29] studied COVID-19 diagnosis from chest CT scans via an ensemble learning method based on ordinal regression. Their proposal relies on multi-binary, neuron stick-breaking, and soft label techniques. Mukherjee et al. [30] implemented an advanced deep network architecture with two CT image datasets. The authors utilized the transfer learning strategy with custom-sized input tailored to each type of deep architecture in order to improve the performance. Their best models achieved an average accuracy of 99.4%.

Nasiri and Hasani [31] proposed a method for diagnosing coronavirus disease from X-rays. They used the DenseNet169 deep neural network (DNN). The extracted features were then used as input for the Extreme Gradient Boosting (XGBoost) algorithm to perform the classification task. They achieved accuracy up to 99.78%. Ullah et al. [32] developed an effective COVID-19 detection technique using the Shufflenet CNN by employing three types of images; i.e., chest radiographs, CT scans, and ECG trace images. Nasiri and Alavi [33] proposed a pretrained network named DenseNet169 to extract features from X-ray images. Analysis of variance (ANOVA) was employed as a feature selection method to reduce the computation and time complexity. Then, the selected features were classified with Extreme Gradient Boosting (XGBoost). Their proposed method reached 98.72% accuracy for two-class classification and 92% accuracy for multiclass classification.

## 3. Proposed Methodology

**Figure 1:**
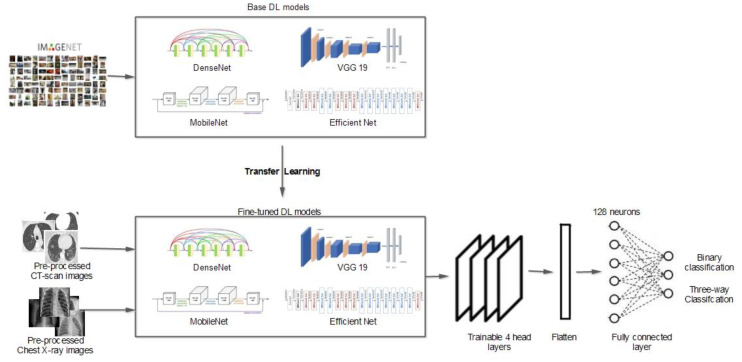
Overall architecture of the proposed methodology.

When building the diagnostic DL model, the chest X-ray and CT images were initially collected. In this study, as illustrated in Figure 1, a publicly available SARS-CoV-2 CT scan dataset was used [34]. The dataset contained 1252 CT scans positive for COVID-19 and 1230 CT scans from non-infected individuals. Another chest X-ray dataset [35] with 6939 sample images was also considered in this study, which included three classes (COVID-19, normal, and pneumonia) with 2313 samples for each category. Section 4.1 provides a detailed description of the datasets utilized. Preprocessing, one of the basic phases in DL learning, is responsible for resizing images to fit the deep learning model. Other processes are also performed to prepare the images for the next phase, such as data augmentation to select the more diverse, more robust datasets to train the model; image grayscale conversion; and image binarization.

Transfer learning is an inevitable step for networks with sparse data (a few hundred or thousand images). Transfer learning is applied to a vast, pretrained network of millions of images. There are two main techniques for applying transfer learning: feature extraction and fine-tuning. For the first technique, only some of the newly added layers are updated and improved during the training phase. In contrast, for the second technique, the weights for all layers are updated, optimized, and customized for the new classification problem. In general, fine-tuning is more effective than the feature extraction technique. Fine-tuning DL models (EfficientB0, VGG-19, DenseNet121, MobileNetV2, etc.) requires extensive resources and time. Initially, the convolution layers learn low-level features and, as the network grows, mid/high-level features are learned. With fine-tuning, these trained low-level features are retained, while the high-level features are trained for new classification problems. In this study, five residual blocks were used: the input, two convolution layers, a max-pooling layer, and an output layer. Subsequently, fine-tuning transfer learning was employed for the first four head layers of the network. The trainable parameters were adjusted along with the supplemented soft-max activation function, which consisted of two or three output neurons relating to binary or three-way classification. Algorithm 1 summarizes the working steps for our DL model.

**Algorithm 1:** DL Model Working StepsInput: *τ*_1_←*Dataset containing SARSCOV2 CT—scans*   *τ*_2_←*Dataset containing chest X—ray images*   *α*←*Learning rate*   *β*←*Batch size*Output: *ω*←*CNN final weights*
**Begin:**
1: Set train and test data sizes2: Calculate train class weights3: Feed in a base model (IMAGENET weights)  // EfficientB0, VGG-19, DenseNet121, EfficientB7, or MobileNetV24: Generate a new model (transfer learning of low layers)5: Set model’s top layers  // average pooling, flatten, dense, dropout6: Set the initial hyperparameters: *α, β, ω*7: Train the base models and store the final weights (*ω*)8: **While** (stopping condition not reached) **do**
9:  Move forward and calculate cross-entropy $E_{c}=-\left(ylog\left(p\right)+\left(1-y\right)\log\left(1-p\right)\right)$10:  Move backward and update the optimizer11: **EndWhile**

In this section, the architectures for the CNNs and the transfer learning approach are described. Although CNNs are more similar to vanilla neural networks, the convolution operation is carried out in more than one layer [36]. A simple neural network layer is presented in Equation (1).

(1) $$z^{\left[1\right]}=g(W^{\left[1\right]}a^{\left[0\right]}+b^{\left[1\right]})$$

where $z^{\left[1\right]}$ is the current layer; $a^{\left[0\right]}$ is the first or input layer; $W^{\left[1\right]}$ represents the weights for the first layer; and $b^{\left[1\right]}$ is the bias. For instance, for the VGG19 Conv layer [37] in Equation (2), for each channel of x, there is a corresponding channel in the first filter of $W_{C}{}^{\left[1\right]}$. Equation (3) illustrates the output of the final layer.

(2) $$z_{\left(i,j,k\right)}^{\left[1\right]}=\left(x*W_{c}^{\left[1\right]}\right)\left(i,j,k\right)+b_{\left(k,1\right)}^{\left[1\right]}$$

(3) $$z_{\left(i,j,k\right)}^{\left[1\right]}=\sum_{\left(l,m,n\right)=1}^{3}W_{c\left(l,m,n,k\right)}^{\left[1\right]}a_{(i+l_{1},j+m_{1,n)}}^{\left[0\right]}+b_{\left(k,1\right)}^{\left[1\right]}$$

where *i*, *j*, and *k* correspond to the row, column, and channel for $z^{\left[1\right]}$, respectively; *l*, *m*, and *n* refer to the row, column, and channel number for the filter, respectively; and *k* denotes the filter being used for the present epoch.

**Figure 2:**
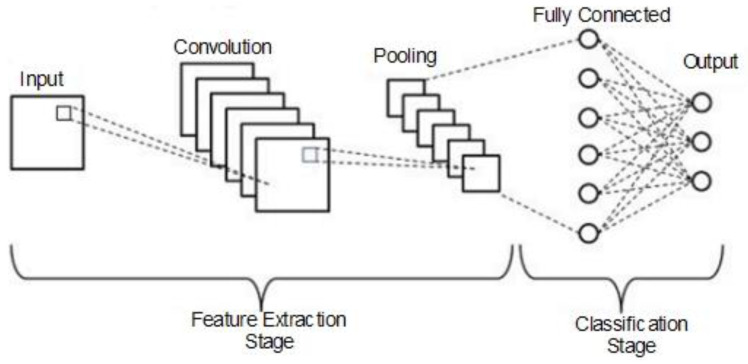
General convolution operation carried out by CNNs.

Figure 2 depicts the general convolution operation carried out by the CNNs, which comprised input, convolution, pooling, fully connected, and output layers. The chest X-ray and CT scan dataset images were fed into the input layer.

**Figure 3:**
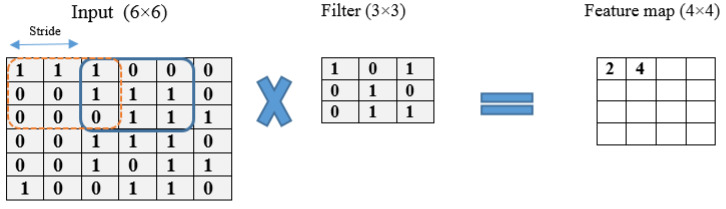
An example of a convolution operation with a resulting 4 × 4 feature map, filter size of 3, and stride of 2.

**Figure 4:**
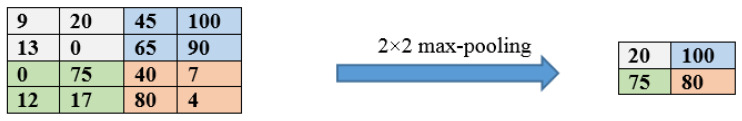
An example of a max-pooling operation with 2 × 2 window size.

**Figure 5:**
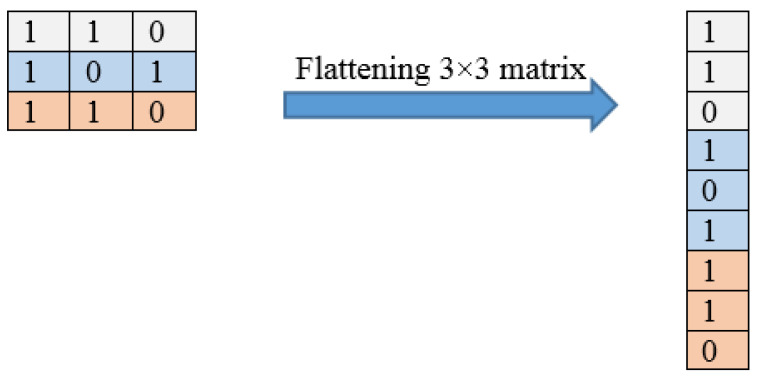
An example of a flattening operation.

Figure 3 illustrates an example of the convolution operation with a 6 × 6 matrix using a stride of 2 and a 3 × 3 filter. The stride value defines the moving filter window of the input matrix. The pooling layer, which comes after the convolutional layer, is responsible for reducing the network computational loss; it is a fully connected layer where all neurons receive their inputs from the flattened form of the previous convolutional layer, as illustrated in Figure 4. An example of a flattening operation is depicted in Figure 5. In our study, some of the popular pooling functions considered were the average, L2 norm, minimum, and maximum functions. In addition, the output layer depends on the number of categories required to train the DL models. In our experiments, two different datasets were utilized: the CT scan dataset, which has a binary classification of COVID and non-COVID; and the chest X-ray dataset, which has a triple classification of COVID-19, normal, and pneumonia classes.

The proposed DL models consider the pretrained weights, which help in learning COVID-19 cases. Three main steps follow: in the first step, the training and test datasets for CT scan or chest X-ray images are prepared. Here, the first CT scan dataset was divided into a training set and testing set, and the training data samples were used to learn the utilized models. The split ratio for the training and testing sets was 978:274 for the COVID-19 class and 1006:223 for the non-COVID class, as presented in Table 1. Further, for the second chest X-ray dataset, there was an approximately equal distribution between COVID-19, normal, and pneumonia classes, with 1850 for training and 463 for testing, as reported in Table 2.

In the second step, the base model and the new model are generated. Here, five main models with weights pretrained with ImageNet were used as the base models. The experiments were run many times with the intention of reaching the most suitable hyperparameters, which, in turn, would provide the best results. Table 3 summarizes the hyperparameters for the different DL models used in this study, and Table 4 presents the characteristics of the DL model architectures used in our experiments. Finally, in the third step, the trained weights are updated and then stored. Hence, once the forward propagation is completed, the binary cross-entropy loss function (Equation (4)) is calculated for the output layer.

(4) $$E_{c}=-\left(ylog\left(p\right)+\left(1-y\right)\log\left(1-p\right)\right)$$

where *y* denotes the true value, and *p* denotes the probability predicted by the model. Then, when the backpropagation process occurs, it counts the number of changes in the weights. Traveling forward and backward is called one epoch, and during one epoch one sample from the dataset is passed per batch size (BS).

## 4. Experimental Results and Discussion 

### 4.1. Description of Datasets

**Figure 6:**
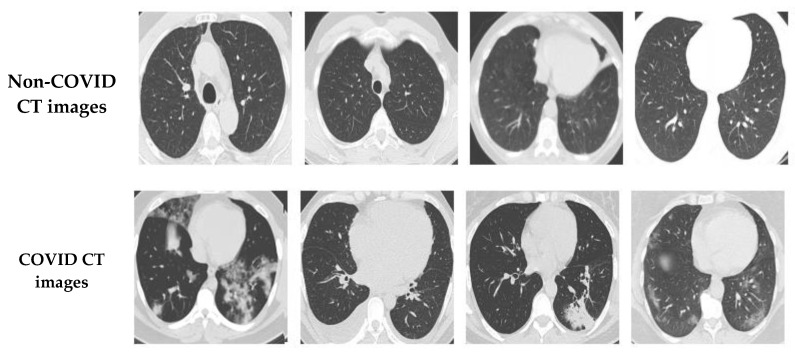
Sample COVID-19 and non-COVID CT images.

**Figure 7:**
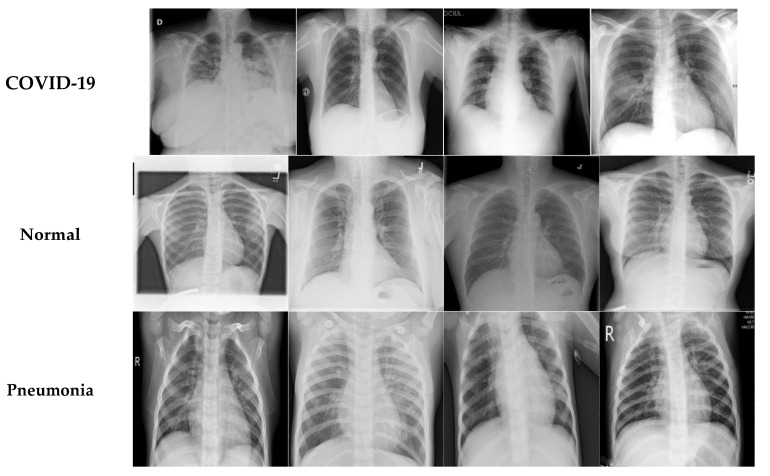
Sample COVID-19, normal, and pneumonia images.

In this study, two different open-access sources were used as our basic experimental datasets. The CT scan dataset [34] had a total number of 2481 CT images divided into 1229 normal cases and 1252 COVID-19 patients, whereas the chest X-ray dataset [35] had 6939 sample images consisting of three classes (COVID-19, normal, and pneumonia), with 2313 samples used for each category. Figure 6 shows samples from the CT scan dataset and Figure 7 shows samples from the chest X-ray dataset. Next, the database was split into training and testing sets. The details for the training and testing samples are shown in Table 1 and Table 2 for the CT scan and chest X-ray datasets, respectively, with the results of the different models displayed in the subsequent tables. Table 1 describes the splitting strategy used for the training and testing sets in the experiment for the CT scan dataset. The 80–20 training–testing ratio was adopted in our experiments. The COVID-19 class had 1013 images for training and 239 images for testing, while the normal class had 971 images for training and 258 for the testing set. Table 2 describes the splitting strategy used for the chest X-ray dataset. This dataset had an equal distribution, with 1850 images for training and 463 images for testing in each class. Data augmentation techniques were employed here to effectively increase the number of training samples. The images were augmented through cropping, noising, brightness modifications, contrast modifications, and random flipping.

### 4.2. Performance Metrics 

In order to measure the performance of the proposed DL models, Equations (5)–(9) were used.

(5) $$Sensitivity=\frac{\mathrm{TP}}{\mathrm{TP}\;+\;\mathrm{FN}\;}$$

(6) $$Specifity=\frac{TN}{TN+FP\;}$$

(7) $$Precision=\frac{TP}{TP+FP\;}$$

(8) $$Accuracy=\frac{TP+TN}{\left(TP+FP+TN+FN\right)\;}\;$$

(9) $$F_{1}=\frac{2*Recall*Precision\;}{Recall+Precision\;\;}$$

### 4.3. Results for DL Models 

Table 5 reports the results obtained for the five baseline DL models (EfficientB0, VGG-19, DenseNet121, EfficientB7, and MobileNetV2) in the classification task for both the CT scan and chest X-ray datasets. The maximum values are marked in red and underlined. Moreover, Table 5 also reports the run time in seconds for the training and testing sets. The minimum measured values are also marked in red and underlined. As presented in the table, for the CT scan dataset, all five models provided an average accuracy greater than 95%, while for the chest X-ray dataset, all models provided an average accuracy greater than 95% except for the EfficientB7 model, which had 89.04% average accuracy.

For the CT scan dataset, EfficientB0 achieved the highest results (99%) in terms of precision and the F1-score, while VGG-19 and DenseNet121 attained the lowest average scores (96%). The MobileNetV2 model achieved the highest averages (99.18% and 99.19) for sensitivity and accuracy, respectively, while EfficientB7 achieved the highest score (99.74%) in terms of specificity. In contrast, VGG-19 attained the lowest averages for sensitivity, specificity, and accuracy. MobileNetV2 achieved the best results in terms of training and testing run times, with 117.43 s for the training run time and 0.77 s for the testing run time, respectively. In general, the MobileNetV2 model can be considered the superior model in comparison to the other four models.

For the chest X-ray dataset, DenseNet121 achieved the highest average precision, F1-score, specificity, and accuracy with 99.57%, 99.56%, 99.78%, and 99.71% respectively. The EfficientB0 model achieved the highest average sensitivity with 99.77%. The VGG-19 model achieved the lowest average values for all metrics. In general, the DenseNet121 model can be considered the best model, despite MobileNetV2 having the lowest training run time. For more details, see Appendix A. 

Table 6 presents the detailed results for the two categories (COVID-19 vs. non-COVID) obtained with the five mentioned DL models with regard to precision, sensitivity, specificity, F1-score, and accuracy. As can be observed in the table, all models achieved accuracy greater than 95%. Regarding the COVID-19 class, the highest values are underlined and marked in red, while the non-COVID class is underlined and marked in green. For the COVID-19 category, the EfficientB0 model was the best in terms of precision and F1-score. However, EfficientB7 achieved the highest sensitivity and specificity, while MobileNetV2 achieved the highest accuracy. Regarding the non-COVID class, the EfficientB0 model was also the best in terms of precision and F1-score. The MobileNetV2 model achieved the highest sensitivity, specificity, and accuracy. For more details, see Appendix A. 

Table 7 presents the detailed results for the three-category (COVID-19 vs. normal vs. viral pneumonia) dataset. The highest values for the COVID-19 class are underlined and marked in red, the highest values for the normal class are underlined and marked in green, and, finally, the highest values for the viral pneumonia class are underlined and marked in blue. As can be observed in the table, for the COVID-19 category, DenseNet121 achieved the highest precision, sensitivity, specificity, F1-score, and accuracy with 98.93%, 99.46%, 100%, 99.45%, and 99.63%, respectively. DenseNet121 also achieved the highest values for the normal class for all metrics with 100%, 99.89%, 99.78%, 100%, and 99.92%, respectively. Furthermore, for the viral pneumonia class, it achieved the best results in terms of precision, specificity, and accuracy with 99.78%, 99.89%, and 99.56%, respectively. However, MobileNetV2 surpassed DenseNet121 in terms of the F1-score and sensitivity, achieving 99.35% and 98.91%, respectively. For more details, see Appendix A. 

**Figure 8:**
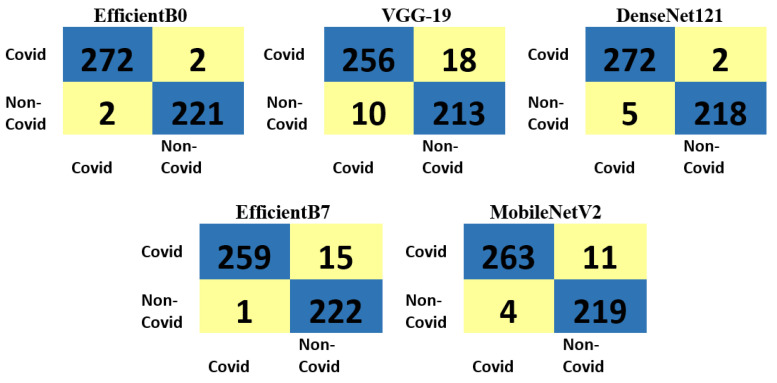
The 80–20% confusion matrixes for the five models with the CT scan dataset.

**Figure 9:**
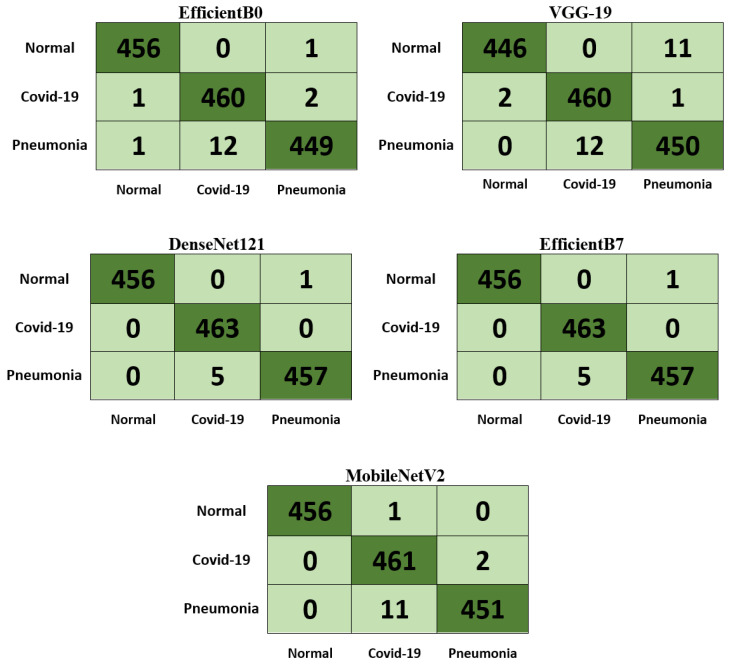
The 80–20% confusion matrixes for the five models with the chest X-ray dataset.

Figure 8 illustrates the 80–20% confusion matrixes for the five models with the CT scan dataset, showing support of 274 and 223 for the COVID-19 and normal classes, respectively. The best model, MobileNetV2, misclassified only 2 out of 274 of the COVID-19 images as normal. Moreover, 2 out of 223 of the normal images were also misclassified. In contrast, the worst model, VGG-19, misclassified ten images in the COVID-19 class as normal. Figure 9 illustrates the 80–20% confusion matrixes for the five models with the chest X-ray dataset, showing support of 457, 463, and 462 for the normal, COVID-19, and viral pneumonia classes, respectively. As can be observed, DenseNet121 was the best model because it classified all COVID-19 images (463 images) accurately. Classifying COVID-19 images accurately with no errors is a remarkable result, as the control is spread among individuals. Moreover, only one normal image was misclassified as pneumonia, and five pneumonia images were misclassified as COVID-19. However, this is less severe than misclassifying COVID-19 images. It can be observed that the DenseNet121 model was not confused with respect to COVID-19 images.

**Figure 10:**
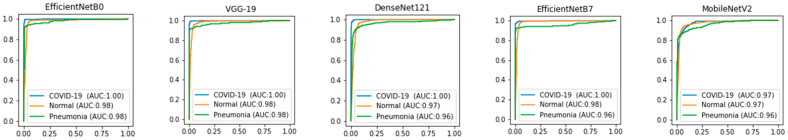
ROC curves for the TPR vs. FPR for chest X-ray dataset images.

**Figure 11:**
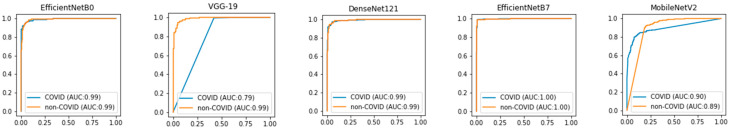
ROC curves for the TPR vs. FPR for CT scan dataset images.

**Figure 12:**
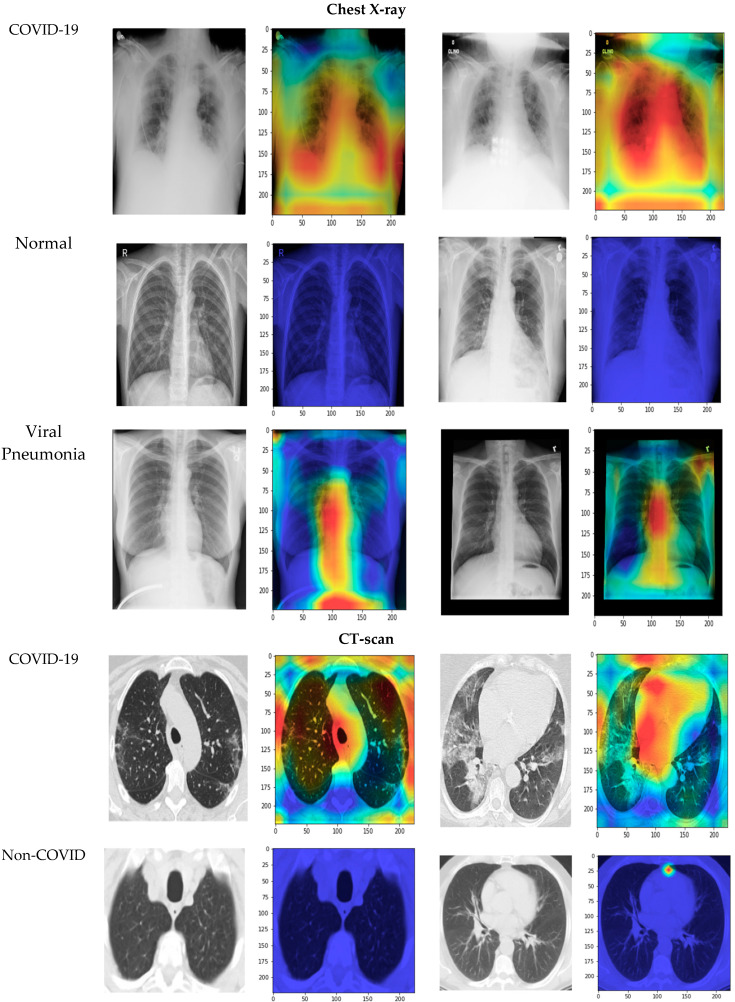
Grad-CAM sample results for COVID-19, normal, and viral pneumonia classes in the chest X-ray dataset and COVID-19 and non-COVID classes in the CT scan dataset.

Figure 10 shows the receiver operating characteristic (ROC) curves for the true-positive rate (TPR) vs. false-positive rate (FPR) for the normal, viral pneumonia, and COVID-19 chest X-ray dataset images. Furthermore, the values of the area under the curve (AUC) are shown in the figure for each category. In addition, Figure 11 shows the ROC curves for the COVID-19 and non-COVID CT scan dataset images. Figure 12 illustrates the results of applying the Grad-CAM algorithm to cover the chest X-ray and CT scan dataset images with heat maps. In the chest X-ray images, the class activation mapping was undertaken by concentrating on particular portions of the normal, viral pneumonia, and COVID-19 classes. In the CT scan images, we applied the Grad-CAM algorithm to COVID-19 and non-COVID classes. In general, in the normal images in both datasets, there was not any kind of opacity that distinguished normal patients from other patients. As depicted in Figure 12, there were no significant localized regions in normal images. In other classes, our models demonstrated the capability to detect the localized regions in the heat maps generated.

### 4.4. Comparative Study

Table 8 presents a comparison of the best proposed DL model and the state-of-the-art method [38] that obtained the best results in the literature for the CT scan dataset. The model proposed here surpassed the state-of-the-art method in terms of sensitivity, specificity, and accuracy. Moreover, the authors of [38] relied on a deep feature fusion stage extracted from the deep features of AlexNet, GoogleNet, ShuffleNet, and ResNet-18. Therefore, their fusion demands an enormous number of parameters, which, in turn, requires excessive processing time.

Table 9 presents a comparison of the best proposed DL model and the state-of-the-art methods for the chest X-ray dataset. The worst results, such as for the model from [34], were obtained with models pretrained for feature extraction instead of with the transfer learning strategy. In general, the results demonstrate the superiority of the proposed model for the chest X-ray three-class classification task, and a remarkably higher average accuracy result of 99.32% was achieved in the case of the DenseNet121 model. Fine-tuning with a moderate number of layers and parameters contributed to these results appreciably. Similarly, dimension reduction promotes faster learning, which was reflected in the short training time (2906.43 s).

## 5. Conclusions

In this study, two primary benchmark datasets for CT and X-ray images were used. All images were enhanced and preprocessed as part of the basic DL learning phase. COVID-19 images were classified as positive or negative using a set of fine-tuned transfer learning models. A set of deep learning models were trained and tested in this research study. For the CT scan dataset, all five models provided average accuracy greater than 95%, whereas, for the chest X-ray dataset, all models provided average accuracy greater than 95% except EfficientB7, which achieved 89.04% accuracy. Compared to the methods in the literature, the results show that MobileNetV2 surpassed the best method in terms of sensitivity, specificity, and accuracy, with a training run time of 117.43 s and testing run time of 0.77 s. In addition, DenseNet121 achieved the highest precision, specificity, F1-score, and accuracy for X-ray images with 99.57%, 99.78%, 99.56%, and 99.71% respectively. In the future, a prediction approach based on a combination of these DL models will be considered in order to improve the results. In addition, more complicated and larger datasets will be used for training to assess the robustness of the proposed approach. Moreover, using CT scans for COVID-19 detection may bring extra radiation to patients. In the future, we will consider the radiation dose issue in our proposed models.

## Figures and Tables

**Table 1:** Frequency of training and testing images in the first dataset (CT scan).

Split	COVID-19	Non-COVID
Training set	978	1006
Testing set	274	223
Total	1252	1229

**Table 2:** Frequency of training and testing images in the second dataset (chest X-ray).

Split	COVID-19	Normal	Viral Pneumonia
Training set	1850	1850	1850
Testing set	463	463	463
Total	2313	2313	2313

**Table 3:** Values for the hyperparameters used in the experiments.

Hyperparameters	Value
Number of epochs	100
Batch size	64
Train–test split ratio	80–20
Optimizer	Adam
Learning rate	1 × 10^−3^
Dropout	0.5

**Table 4:** Characteristics of the DL model architectures used in the experiments.

Model	Default Input Size	Custom Size	No. of Layers	No. of Parameters
EfficientB0	224 × 224	64 × 64	237	04,384,248
VGG-19	224 × 224	64 × 64	19	20,159,382
DenseNet121	224 × 224	64 × 64	121	07,305,622
EfficientB7	224 × 224	64 × 64	813	64,765,158
MobileNetV2	224 × 224	64 × 64	53	02,592,662

**Table 5:** Average classification results obtained for both the CT scan and chest X-ray datasets.

Model/Metric	Precision	Sensitivity	Specificity	F1-Score	Accuracy	Train Time (S)	Test Time (S)
**CT-Scan Dataset**							
**EfficientB0**	99.00%	98.06%	98.06%	99.00%	98.18%	172.49	1.38
**VGG-19**	96.00%	95.93%	95.93%	96.00%	95.97%	194.88	0.19
**DenseNet121**	96.00%	97.92%	97.92%	96.00%	97.98%	242.62	1.75
**EfficientB7**	98.00%	98.82%	99.74%	98.00%	98.79%	866.35	4.88
**MobileNetV2**	98.00%	99.18%	99.18%	98.00%	99.19%	117.43	0.77
**Chest X-Ray Dataset**							
**EfficientB0**	98.78%	99.77%	99.38%	98.77%	99.17%	2887.13	29.50
**VGG-19**	98.13%	98.11%	99.05%	98.12%	98.74%	3108.95	26.07
**DenseNet121**	99.57%	99.56%	99.78%	99.56%	99.71%	2906.43	32.16
**EfficientB7**	84.76%	83.55%	91.77%	83.79%	89.04%	2880.74	24.54
**MobileNetV2**	99.00%	98.98%	99.49%	98.99%	99.32%	2107.61	58.57

**Table 6:** Classification results of the five DL models for the two-category (COVID-19 vs. normal) CT scan dataset.

Model	Category	Precision	Sensitivity	Specificity	F1-Score	Accuracy
**EfficientB0**	COVID-19	99.00%	99.27%	96.86%	99.00%	98.18%
	Non-COVID	99.00%	96.86%	99.27%	99.00%	98.18%
**VGG-19**	COVID-19	96.00%	96.35%	95.51%	96.00%	95.97%
	Non-COVID	96.00%	95.51%	96.35%	95.00%	95.97%
**DenseNet121**	COVID-19	96.00%	98.54%	97.30%	96.00%	97.98%
	Non-COVID	96.00%	97.30%	98.54%	96.00%	97.98%
**EfficientB7**	COVID-19	97.00%	98.54%	99.26%	98.00%	98.79%
	Non-COVID	99.00%	99.10%	98.22%	98.00%	98.79%
**MobileNetV2**	COVID-19	98.00%	99.27%	99.10%	98.00%	99.19%
	Non-COVID	98.00%	99.10%	99.27%	98.00%	99.19%

**Table 7:** Classification results for the five DL models for the three-category (COVID-19 vs. normal vs. viral pneumonia) chest X-ray dataset.

Model	Category	Precision	Sensitivity	Specificity	F1-Score	Accuracy
**EfficientB0**	COVID-19	97.46%	99.35%	98.69%	98.40%	98.91%
	Normal	99.56%	99.78%	99.78%	99.67%	99.78%
	Viral pneumonia	99.34%	97.18%	99.67%	98.25%	98.84%
**VGG-19**	COVID-19	97.46%	99.35%	98.69%	98.40%	98.91%
	Normal	99.55%	97.59%	99.78%	98.56%	99.05%
	Viral pneumonia	97.40%	97.40%	98.69%	97.40%	98.26%
**DenseNet121**	COVID-19	98.93%	100%	99.45%	99.46%	99.63%
	Normal	100%	99.78%	100%	99.89%	99.92%
	Viral pneumonia	99.78%	98.91%	99.89%	99.35%	99.56%
**EfficientB7**	COVID-19	72.76%	85.96%	83.78%	78.81%	84.51%
	Normal	92.58%	79.21%	96.86%	85.38%	91.02%
	Viral pneumonia	88.96%	85.49%	94.67%	87.20%	91.60%
**MobileNetV2**	COVID-19	97.46%	99.56%	98.69%	98.50%	98.98%
	Normal	100%	99.78%	100%	99.89%	99.92%
	Viral pneumonia	99.56%	97.61%	99.78%	98.58%	99.05%

**Table 8:** The results obtained with the CT scan dataset compared to state-of-the-art methods.

Authors/Year	Method	Precision	Sensitivity	Specificity	F1-Score	Accuracy	Drawbacks
Silva et al. [25]/2020	EfficientNet family	93.51%	79.59%	93.98%	86.19%	87.60%	Low accuracy
Singh et al. [39]/2020	Multi-objective differential evolution (MODE) model	N/A	90.50%	90.50%	N/A	93.00%	Low accuracy
Panwar et al. [22]/2020	VGG19 model	N/A	94.04%	95.84%	N/A	95.00%	High computational complexity
Jaiswal et al. [2]/2021	DenseNet121	96.29%	96.29%	96.21%	96.29%	96.25%	Use of a dataset with a limited number of samples
Dina Ragaband Omneya Attallah [38]/2020	FUSI-CAD	99.00%	99.00%	99.00%	99.00%	99.00%	High computational complexity
This work	MobileNetV2	98.00%	99.18%	99.18%	98.00%	99.19%	Does not use hyperparameter optimization (HPO) algorithms

**Table 9:** The results obtained for the chest X-ray dataset compared to the state-of-the-art methods.

Authors/Year	Method	Precision	Sensitivity	Specificity	F1-Score	Accuracy	Drawbacks
Ozturk et al. [40]/2020	DarkCovidNet	89.96%	85.35%	N/A	87.37%	87.02%	Low accuracy
Apostolopoulos and Mpesiana [41]/2020	VGG-19	92.85%		N/A		93.48%	Low accuracy
Agrawal et al. [10]/2021	FocusCovid	95.60%	95.20%	N/A	95.20%	95.20%	Use of a dataset with a limited number of samples
Cengil et al. [4]/2022	AlexNet + EfficientNet-B0 + N2ASNetLarge+ Xception and SVM	99.80%	98.60%	99.90%	99.19%	95.70%	High computational complexity
Ouchicha et al. [42]/2020	CVDNet	96.72%	96.84%	N/A	96.68%	96.69%	High computational complexity
Nasiri and Alavi [31]/2022	DenseNet169 +ANOVA + XGBoost	99.02	93.18	100	97.57	98.72	Sensitivity to the number of features selected by the ANOVA
Nasiri et al. [12]	DenseNet169 +MobileNet +LightGBM	95.12	95.20	97.16	95.60	98.54	Dependence on only the patient’s chest X-ray
This work	DenseNet121	99.00%	98.98%	99.49%	98.99%	99.32%	Does not use hyperparameter optimization (HPO) algorithms

## Data Availability

The data used to support the findings of this study are available at: https://www.medrxiv.org/content/10.1101/2020.04.24.20078584v3 (15 March 2022) for the CT dataset and: https://data.mendeley.com/datasets/mxc6vb7svm/1 (15 March 2022) for the chest X-ray dataset.

## Appendix A

Two-class experiment results.

**Table A1:** EfficientB0.

Iteration	Precision	Sensitivity	Specificity	F1-Score	Accuracy	Train Time (S)	Test Time (S)
1	98.5	97.81	99.1	98.5	98.39	172.603498	0.190268278
2	96.5	96.72	99.1	98	97.79	172.845818	0.180725336
3	99.5	99.27	100	100	99.6	172.845818	0.180725336
4	99	97.81	99.55	98.5	98.59	171.685683	0.164677143
5	98	97.81	98.21	98	97.99	170.939578	0.156193256
Average	98.3	97.884	99.192	98.6	98.472	172.765045	0.17451787
STDEVP	1.029563	0.8114579	0.593680049	0.734847	0.6309485	0.11423032	0.012310743

**Table A2:** VGG-19.

Iteration	Precision	Sensitivity	Specificity	F1-Score	Accuracy	Train Time (S)	Test Time (S)
1	94.5	93.8	95.07	94.5	94.37	194.469967	0.215785742
2	88	98.54	76.6	89.5	88.73	195.344422	0.228986979
3	88.5	82.12	95.07	88	87.93	194.913571	0.211827993
4	94.5	91.97	97.31	94.5	94.37	195.879034	0.212376118
5	62	100	61.43	65.5	65.39	194.292557	0.216182947
Average	85.5	93.286	85.096	86.4	86.158	194.97991	0.217031956
STDEVP	12.078907	6.314395	14.00289056	10.77219	10.732557	0.57934254	0.006228234

**Table A3:** DenseNet121.

Iteration	Precision	Sensitivity	Specificity	F1-Score	Accuracy	Train Time (S)	Test Time (S)
1	99	97.81	100	99	98.79	172.484457	0.215648413
2	97.5	96.35	98.65	97	97.38	248.973759	0.233531952
3	96.5	96.35	96.86	96.5	96.58	250.652627	0.235277414
4	95.5	90.88	100	95	94.97	241.603891	0.249029398
5	97	97.08	96.86	97	96.98	240.935437	0.248332977
Average	97.1	95.694	98.474	96.9	96.94	230.930034	0.236364031
STDEVP	1.1575837	2.4671327	1.40700533	1.280625	1.235168	29.4770471	0.012181999

**Table A4:** EfficientB7.

Iteration	Precision	Sensitivity	Specificity	F1-Score	Accuracy	Train Time (S)	Test Time (S)
1	98.5	98.9	98.21	98.5	98.58	857.0554817	0.78863883
2	97.5	99.64	96.86	97.5	98.3	868.8623106	0.829064846
3	98.5	96.72	99.55	98	98	866.0339637	0.782578707
4	98	99.64	96.41	98	98.19	860.6685259	0.793141842
5	97.5	99.27	95.52	98	97.59	870.467566	0.783352852
Average	98	98.834	97.31	98	98.132	864.6175696	0.795355415
STDEVP	0.4472136	1.0920366	1.417335528	0.316228	0.3296908	5.040368568	0.017283772

**Table A5:** MobileNetV2.

Iteration	Precision	Sensitivity	Specificity	F1-Score	Accuracy	Train Time (S)	Test Time (S)
1	95	93.43	96.86	95	94.97	115.561078	0.133627653
2	91.5	89.42	94.17	91.5	9155	116.526959	0.135413647
3	92	96.72	88.79	93.16	93.16	118.17297	0.113469124
4	93	98.54	86.55	93.16	93.11	119.257729	0.146316767
5	80.5	80	91.48	85.5	85.11	116.967638	0.188757896
Average	90.4	91.622	91.57	91.664	1904.27	117.297275	0.143517017
STDEVP	5.0931326	6.5884274	3.679157512	3.274957	3625.3666	1.29047647	0.024983658

Three-class experiment results.

**Table A6:** EfficientB0.

Iteration	Precision	Sensitivity	Specificity	F1-Score	Accuracy	Train Time (S)	Test Time (S)
1	95.33	98.19	99.34	95.33	96.878002	447.695684	0.437743187
2	95.33	98.87	98.9	95.33	96.829971	432.421255	0.447622299
3	95	99.09	98.88	95	96.637848	425.374457	0.467562675
4	96.5	99.09	98.69	96.5	97.118156	431.332181	0.417729855
5	95	97.96	98.67	95	96.589817	425.298462	0.444066763
Average	95.432	98.64	98.896	95.432	96.810759	435.163799	0.442944956
STDEVP	0.5540181	0.4738776	0.241213598	0.554018	0.1887309	9.31666799	0.016074998

**Table A7:** VGG-19.

Iteration	Precision	Sensitivity	Specificity	F1-Score	Accuracy	Train Time (S)	Test Time (S)
1	96.3	99.09	99.13	96.3	97.358309	520.112408	0.527137995
2	96	98.87	98.7	96	97.358309	513.283731	0.539829969
3	95.3	99.77	98.61	95.6	96.829971	508.651526	0.510110378
4	96.6	99.55	99.34	96.3	97.646494	514.29095	0.525996685
5	96.3	99.32	98.7	96.6	97.646494	510.08004	0.54573822
Average	96.1	99.32	98.896	96.16	97.367915	513.283731	0.52976265
STDEVP	0.4427189	0.3196248	0.286537956	0.338231	0.298255	3.98412533	0.012360039

**Table A8:** DenseNet121.

Iteration	Precision	Sensitivity	Specificity	F1-Score	Accuracy	Train Time (S)	Test Time (S)
1	96.6	99.1	99.13	96.6	97.646494	562.344443	0.638468742
2	96	100	98.9	96.3	97.214217	547.1	0.624609947
3	96.6	99.77	98.91	96.3	97.502402	542.371586	0.577787161
4	96	99.55	99.12	96.3	97.214217	542.873865	0.610474825
5	95.6	98.64	99.13	96	97.166186	540.810979	0.623207569
Average	96.16	99.412	99.038	96.3	97.348703	547.100175	0.614909649
STDEVP	0.3878144	0.4870893	0.108701426	0.189737	0.1906766	7.90142936	0.020570508

**Table A9:** EfficientB7.

Iteration	Precision	Sensitivity	Specificity	F1-Score	Accuracy	Train Time (S)	Test Time (S)
1	96	99.32	98.48	96	97.358309	2354.27585	2.09230423
2	96.3	98.19	98.92	96.3	97.454371	2337.126517	2.091997385
3	95.3	97.72	99.13	96.3	97.070125	2339.053592	2.10710454
4	93.3	97.2	99.11	95.3	95.917387	2329.031113	2.064648151
5	95.6	98.42	98.92	96.3	97.070125	2326.145514	2.092447042
Average	95.3	98.17	98.912	96.04	96.974063	2337.126517	2.08970027
STDEVP	1.056409	0.7111681	0.23387176	0.387814	0.5501564	9.838000281	0.013784915

**Table A10:** MobileNetV2.

Iteration	Precision	Sensitivity	Specificity	F-Score	Accuracy	Train Time (S)	Test Time (S)
1	89.66	98.46	98.34	98.6	93.17963497	314.252928	0.366166115
2	92.6	97.62	98.64	92.6	95.14889529	308.500018	0.305682182
3	93	92.74	99.13	92.6	95.77329491	308.500018	0.322913408
4	93.6	97.94	99.53	93.3	95.43707973	308.947497	0.323650837
5	93	99.54	94.16	92.3	95.05283381	301.514411	0.304165363
Average	92.372	97.26	97.96	93.88	94.91834774	308.342975	0.324515581
STDEVP	1.393103	2.3520544	1.943224125	2.382771	0.904917491	4.04899008	0.022391561
